# Understanding Risky Behaviors Among Fishers in a Lower Middle Income Country: A Qualitative Study of Fishers in Elmina, Ghana

**DOI:** 10.1002/puh2.70282

**Published:** 2026-06-15

**Authors:** Sylvester Kyei‐Gyamfi, Frank Kyei‐Arthur, Bismark Bless Nyadzi, Oppong Kwarteng Hammond, Eunice Kisiwaa Gyan, Joha Issaka Braimah

**Affiliations:** ^1^ Department of Children Ministry of Gender, Children and Social Protection Accra Ghana; ^2^ Department of Environment and Public Health University of Environment and Sustainable Development Somanya Ghana; ^3^ Public Affairs Unit Ministry of Gender, Children and Social Protection Accra Ghana; ^4^ General Administration University of Environment and Sustainable Development Somanya Ghana; ^5^ Kofi Annan International Peacekeeping Training Centre Accra Ghana; ^6^ Centre for Migration Studies University of Ghana, Legon Accra Ghana

**Keywords:** Elmina, fishers, Ghana, human immunodeficiency virus (HIV)/acquired immunodeficiency syndrome (AIDS), risk compensation theory, risky behaviors

## Abstract

Migration and the fishing environment heighten fishers’ risk of engaging in risky behaviors, such as substance abuse, engagement with multiple sexual partners, and unprotected sexual activity. This qualitative study examines risky behaviors and the factors contributing to these behaviors among fishers in Elmina, Ghana, through the lens of the risk compensation theory (RCT). The study involved a total sample of 50 participants, comprising 30 key informants (KIIs) and 20 focus group discussion (FGD) participants. Transcripts were analyzed thematically following four stages. The study found that fishers engage in three risky behaviors as follows: excessive alcohol consumption, excessive smoking, and unsafe sexual practices. Moreover, three factors contributed to the engagement of risky behaviors by fishers as follows: substance abuse, spendthrift habits and lavish social interactions, and perceiving fishing as a risky occupation led to discounting the dangers of human immunodeficiency virus (HIV). This study's findings underscore the need for holistic interventions that address not just individual behaviors but also the broader environmental, occupational, and socioeconomic contexts that shape the decision‐making of fishers.

## Graphical abstract

Fishers in Elmina, Ghana, engage in alcohol use, smoking, and unsafe sexual practices shaped by mobility, economic pressures, and risk perceptions. Using risk compensation theory, this study shows how occupational risk normalizes health risk‐taking, highlighting the need for integrated behavioral and structural interventions in fishing communities.

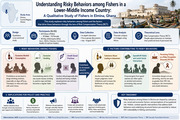

## 1 Introduction

Fishing is an occupation characterized by significant mobility, with male fishers often traveling extensively in search of abundant catches and female fishers following to purchase and sell the catch [1, 2, 3, 4, 5]. This constant movement exposes both male and female fishers to environments that may encourage risky behaviors, particularly in transient and high‐risk coastal communities [3, 4]. Risky behaviors are defined as actions that increase the likelihood of disease or injury, potentially leading to disability, death, or social problems [6]. In the fishing industry, such behaviors often manifest through unprotected sexual activity, engagement with multiple sexual partners, substance abuse, and limited adherence to health and safety protocols [4, 7, 8].

Research from developing countries highlights the link between mobility within the fisheries sector and the spread of sexually transmitted infections (STIs), including human immunodeficiency virus (HIV) [3, 9, 10, 11]. For instance, in communities along Lake Victoria in Kenya, high rates of HIV have been reported among fishers, attributed to the prevalent “jaboya” system, where women exchange sex for fish [3, 9]. Similar patterns are observed in Cambodia's Tonle Sap Lake region, where fishers’ mobility and transactional sex contribute to elevated STI risks [12]. Studies indicate that fishers and residents of fishing communities often exhibit HIV infection rates 5 to 10 times higher than those in the general population [13, 14, 15, 16]. Kishamawe [17] explains that mobility contributes to changing sexual behaviors that increase HIV risk among mobile populations. In Ghana, the situation is similarly concerning, particularly in Elmina, where fishers’ mobility, coupled with economic vulnerabilities, drives engagement in transactional and casual sex [4, 18, 19, 20].

However, although existing studies, including earlier work by Kyei‐Gyamfi [4, 19], have extensively documented the prevalence and correlates of risky behaviors in fishing communities, they remain largely descriptive and provide limited insight into the underlying behavioral mechanisms that shape how fishers make decisions about risk. This study is significant because it moves beyond descriptive epidemiological accounts to provide a behavioral explanation of risky practices among fishers. Specifically, it examines how fishers actively interpret and negotiate competing risks, particularly the trade‐off between immediate occupational hazards (such as the dangers of fishing) and the longer term risk of HIV infection. This focus on competing risk perception represents a critical and underexplored dimension in the literature on fisher communities and HIV/acquired immunodeficiency syndrome (AIDS) in Africa.

The elevated HIV prevalence and mortality rates from AIDS in fishing communities have led to their classification as high‐risk groups requiring special attention in HIV/AIDS prevention efforts [21, 22]. The Ghana AIDS Commission, for example, has identified fishing communities like Elmina as priority areas for intervention, emphasizing the need for targeted education and testing services. Factors contributing to this heightened risk include the high mobility of fishers, leading to prolonged absences from home and increased likelihood of engaging in risky sexual behaviors, such as casual partnerships at new fishing sites [16, 23, 24, 25, 26]. Demographic profiles suggest that many fishers are young, unmarried, and sexually active, with disposable income that may be spent on alcohol and transactional sex, further increasing their risk [3, 8, 16, 23, 25, 26]. Additionally, some fishers exhibit HIV risk denial attitudes, perceiving fishing as a more immediate danger than HIV/AIDS [21, 27].

The Elmina fishing community exemplifies these challenges. Many fishers operate under tight schedules, leading to prolonged periods away from their families and fostering isolation. Such isolation often drives them to seek comfort through alcohol consumption and casual sexual encounters [8, 28, 29]. Furthermore, traditional practices, such as seasonal festivals and communal rituals, which often involve heavy drinking and socialization, contribute to the normalization of risky behaviors. The transient nature of fishing work provides fishers with considerable autonomy, usually leading to engagement in risky behaviors due to the absence of familial oversight [2, 20, 29]. The demanding work schedules and frequent travel associated with fishing can strain social interactions and contribute to feelings of loneliness. For instance, mobile fishers are susceptible to loneliness due to prolonged absences from home and operations across multiple communities without family support [1, 4, 30].

Kirby et al. [31] observe that HIV programs implemented around the world have yielded significant results in improving HIV knowledge and practices, especially in developing countries. However, this is not the case with mobile people, such as fishers. Literature on fishers has shown poor HIV knowledge, attitudes, and practices. These studies have associated poor knowledge, attitudes, and practices with the high mobility of fishers [3, 14, 23, 32]. Pan [33] and Owoaje [34] have also reported that mobile workers’ knowledge of HIV and AIDS is minimal owing to their inability to obtain useful information, consultation, and healthcare services pertaining to HIV and AIDS. Duwal et al. [23] explain that due to their high mobility, fishers hardly stay at one point, making it difficult to reach them with SRHR programs. Additional explanations for poor knowledge include the fact that most fishers have poor educational backgrounds, with the majority being illiterate or school dropouts [23].

Despite evidence linking fishing communities to risky behaviors, there is limited literature detailing the specific justifications for these behaviors among fishers. This study addresses this gap by providing new, context‐specific empirical evidence from HIV hotspot fishing communities in Ghana, thereby contributing geographically grounded insights to the broader African literature on fishers and HIV/AIDS. Furthermore, the study makes a novel theoretical contribution by applying a behavioral (Rational Choice Theory) perspective to understand when, why, and under what conditions fishers engage in or reduce risky behaviors. By integrating behavioral, economic, and occupational dimensions, the study offers new empirical and analytical insights into how risk is actively negotiated in everyday life within fishing communities.

The study sought to answer the following questions: (a) What risky behaviors do fishers in Elmina engage in? and (b) what factors contribute to fishers’ engagement in these risky behaviors? By integrating behavioral, economic, and occupational dimensions, the study provides new empirical insights into how risk is negotiated within HIV hotspot fishing communities. The findings of this study will aid policymakers and health practitioners in comprehensively understanding fishers’ risky behaviors and the factors driving these behaviors. This knowledge is crucial for developing targeted interventions that address the unique challenges faced by fishers, ultimately aiming to reduce the incidence of HIV/AIDS and promote safer practices within these communities.

### 1.1 Theoretical Framework

This study uses risk compensation theory (RCT) to examine the risky behaviors and the factors contributing to these behaviors among Elmina fishers. Importantly, RCT was used a priori to inform the formulation of the research questions, the design of data collection tools, and the analytical focus of the study, rather than being introduced only at the interpretation stage. In the 1980s, Gerald J. S. Wilde, a Canadian psychologist, proposed that humans have a certain threshold of risk that they are comfortable with. When safety precautions are implemented, people tend to change their behavior to retain their preferred level of risk, often engaging in riskier activities when they feel safer. The argument first gained traction in traffic safety disputes; when cars were equipped with improved brakes or seatbelts, drivers would sometimes drive faster or less carefully because they felt more secure. This concept was later applied to other areas, such as health behavior, sports, and sexual risk‐taking. According to the RCT, people modify their behavior based on their perceived level of risk, often increasing hazardous behavior when they feel more protected and lowering it when they sense greater vulnerability [35]. Guided by RCT, this study specifically sought to examine how fishers perceive, prioritize, and respond to different forms of risk in their everyday lives. This theoretical orientation directly informed the development of interview guides and the framing of questions around risk perception, decision‐making, and behavioral adjustment.

In this study, RCT is applied not only as a descriptive lens but as an analytical framework to examine how, when, and under what conditions fishers adjust their behaviors. Specifically, attention is given to three interrelated mechanisms: (a) perceived substitution between occupational and health risks, (b) behavioral adjustment in response to situational cues, such as income gains or social events, and (c) temporal fluctuations in risk‐taking across fishing cycles and mobility patterns. These mechanisms were specified prior to data collection as sensitizing concepts and were used to guide both data generation and thematic analysis.

In fishing villages like Elmina, where occupational hazards like ship accidents are viewed as more immediate concerns than HIV infection, fishers may engage in compensatory behaviors that increase their health risks. For example, the physical risks involved with fishing can normalize risk‐taking behaviors, making HIV/AIDS worries appear less important [21, 27]. As a result, fishers may underestimate the dangers of unprotected sex, many connections, and substance abuse, dismissing them as trivial or unavoidable features of their temporary lifestyles. This reflects a process of perceived risk substitution, where immediate survival risks at sea take precedence over long‐term health considerations.

A major strength of RCT is its realistic portrayal of human behavior; it acknowledges that people's actions are influenced more by perceived risk than actual risk, providing a compelling explanation for why increased HIV knowledge alone may not lead to safer sexual practices. In this study, this assumption was treated as a guiding proposition and examined empirically through participants’ narratives. In this context, the hypothesis explains why some fishermen, despite being aware of HIV/AIDS and having access to preventive instruments, such as condoms, continue to engage in risky sexual activities. The feeling of being “healthy” or a false sense of security following a single negative HIV test result can impair motivation to engage in continuous protective practices. Similarly, if not adequately planned, awareness campaigns and health interventions may unintentionally foster a sense of invulnerability, leading to behavioral disinhibition. In addition, periods of financial gain or successful fishing expeditions may create situational perceptions of safety and reward, thereby increasing the likelihood of engaging in compensatory behaviors, such as alcohol use and unprotected sex.

However, a significant weakness of RCT is its emphasis on individual behavior at the expense of bigger structural variables. Risk perception is subjective and difficult to fully assess because not everyone responds to risk in the same way. In Elmina, the socioeconomic context, which includes poverty, limited alternative livelihoods, and gendered power dynamics in transactional sex relationships, has a substantial impact on the conduct of fishers. By focusing primarily on personal risk assessment, RCTs risk underestimating how these systemic elements influence or constrain individual decisions. To address this limitation, the study integrates RCT with contextual and structural considerations, ensuring that behavioral interpretations are grounded in the lived realities of fishers. This study therefore situates RCT within these broader structural conditions, demonstrating that risk compensation among fishers is not purely individual but is shaped by economic pressures, mobility patterns, and social norms.

Thus, although the theory provides useful insights into behavioral patterns, it must be supplemented by a more comprehensive understanding of the economic and social realities that fishermen face. Applying RCT to the experiences of Elmina's fishermen leads to a better understanding of the intricate interplay between occupational risk, perceived health concerns, and behavioral responses. Overall, RCT was used as an organizing framework throughout the study, from conceptualization and data collection to analysis and interpretation, rather than being applied retrospectively. The study extends the application of RCT by empirically illustrating its mechanisms and testing its relevance within a sub‐Saharan African fishing context, thereby addressing gaps in previous descriptive applications of the theory. It emphasizes the significance of developing HIV/AIDS interventions that not only raise awareness but also actively challenge underlying risk attitudes, promote long‐term behavior change, and address the socioeconomic factors impacting fishers’ decision‐making. Despite its shortcomings, RCT provides a compelling lens for evaluating the dichotomy between knowledge and action among fishers. It also serves as a foundation for designing more effective, context‐sensitive health promotion methods in fishing communities.

## 2 Methods

### 2.1 Study Area

Elmina, located in the Komenda Edina Eguafo Abirem Municipal District in the Central Region of Ghana, was selected for its high concentration of fishers and its status as a prominent fishing hub [36]. The town's bustling fishing activities and social interactions offered a rich context to examine how community norms and practices contribute to risky behaviors. Moreover, Elmina has rich historical sites like the Elmina Castle, which promotes tourism in the community [37, 38].

### 2.2 Study Design and Sampling Procedure

A qualitative cross‐sectional design was adopted to provide an in‐depth understanding of the social and cultural factors influencing risky behaviors among Elmina's fishers. This design was effective in capturing detailed narratives from participants, offering a nuanced perspective on their daily lives and the sociocultural determinants of their health behaviors, including risky behavior, and is widely recommended for exploring lived experiences and social processes within specific contexts [39, 40]. The study prioritized qualitative data to explore not just what behaviors occurred but also why and how they were rationalized within the community.

Purposive sampling was used to select fishers, officials of government and nongovernmental organizations (NGOs), and community opinion leaders with relevant knowledge about fishers’ risky behaviors, ensuring the inclusion of diverse perspectives as recommended for identifying information‐rich cases in qualitative research [39, 41]. This method enhanced the richness of the data by capturing a wide range of views of different stakeholders.

To strengthen sampling rigor and address concerns regarding selection procedures, the qualitative sampling was informed by a prior mapping and listing exercise of fishers across 10 fishing associations in Elmina, which produced a sampling frame of 690 registered members. This approach aligns with established practices for constructing sampling frames in field‐based studies where population lists are not readily available [42]. The associations comprised diverse occupational groups, including canoe fishers, fishmongers, porters, and traders, thereby ensuring variation across the fisheries value chain.

Fishers selected for the focus group discussions (FGDs) were recruited from 10 fishing associations in Elmina. Participants were purposively drawn from this structured sampling frame to ensure diversity in gender, roles, and occupational categories, thereby enhancing the credibility and transferability of the findings [43]. FGD was conducted separately for male and female fishers to explore their perspectives. This approach is consistent with qualitative research practices that seek to capture gendered experiences and encourage open discussion among participants [40]. In total, two FGDs were conducted. Each FGD was made up of 10 participants (Table 1).

**TABLE 1:** Participants of focus group discussions (FGDs).

Type of group	Number of participants
Male	10
Female	10
**Total**	**20**

Regarding the key informant interviews (KIIs), 30 KIIs with officials of government and NGOs and community opinion leaders were selected (Table 2).

**TABLE 2:** Participants of the key informant interviews.

Subject	Frequency
KEEA Administration Office	4
Municipal Health Directorate	1
Ghana AIDS Commission	1
NGOs/CBOs	2
Department of Fisheries	2
Community members/opinion leaders	20
**Total**	**30**

Abbreviations: AIDS, acquired immunodeficiency syndrome; NGOs, nongovernmental organizations.

Key informants were purposively selected on the basis of their roles, experience, and direct engagement with fishing communities and HIV‐related issues. KIIs are widely used to obtain expert and insider perspectives on complex social and health issues [44]. To ensure diversity, participants were drawn from multiple sectors, including local government, health services, fisheries management, civil society, and community leadership, thereby strengthening triangulation of data sources [40].

### 2.3 Data Collection

The data collection occurred between July and August 2017. The study covers various topics, such as fishers’ living arrangements, health behaviors (including risky behaviors), HIV knowledge, and participation in HIV sensitization activities. However, this study focused on fishers’ risky behaviors. In‐depth interviews were conducted with key informants, whereas discussions were held with FGD participants using interview guides. The use of semi‐structured interview guides is consistent with qualitative research approaches that allow flexibility while maintaining focus on key themes [39].

All interviews and discussions were audio‐recorded, and on average, KIIs lasted for 40 min, whereas FGD lasted 75 min. All interviews and discussions were conducted in English and local languages (Fante and Twi) on the basis of the preferences of participants, which enhances data quality and participant comfort [40].

In addition, a pretest was conducted in a fishing community in Accra called Chorkor, which has similar characteristics to Elmina, which helped to refine the questions in the interview guides in line with recommended practices for improving validity and clarity of research instruments [42].

Participation in the study was voluntary, and all participants gave their written informed consent before participating in the KIIs and FGDs. Interviews were conducted in venues convenient for participants, such as offices, homes, and community centers. The study was approved by the Ethics Committee for the Humanities at the University of Ghana. Furthermore, the study adhered to all ethical standards during the data collection, including confidentiality, anonymity, and voluntary participation [39].

### 2.4 Data Analysis

Thematic analysis was used to analyze the transcripts of KIIs and FGDs, which followed four stages. This approach is consistent with the widely used framework for thematic analysis developed by Virginia Braun and Victoria Clarke [45].

In the first stage, the second author read all the transcripts twice to understand the general picture of the participant's perspectives. In the second stage, the narratives of participants in each transcript that talked about the research questions were assigned codes by the second author. The second author also merged codes that were related to similar issues to form themes in the third stage. In the fourth stage, the themes were used to report the results or findings of the study.

To strengthen analytical rigor, reflexivity was maintained throughout the process, with the researchers critically reflecting on their assumptions and potential influence on data interpretation [40].

NVivo qualitative data analysis software was used to support coding, organization, and retrieval of data, thereby enhancing transparency and consistency in the analysis [46].

To ensure the trustworthiness of the findings, all co‐authors reviewed the themes and their codes generated by the second author to ensure they reflect the perspectives of participants as well as ensure the themes are derived from their corresponding codes. In addition, the study adhered to established criteria of credibility, dependability, and confirmability to enhance trustworthiness [43]. Moreover, all decisions made during the study and analysis were recorded for transparency and replications.

## 3 Results

### 3.1 Sociodemographic Characteristics of Key Informants

Table 3 presents the sociodemographic characteristics of key informants. Table 3 shows that two‐thirds of key informants were males (66.7%) and had formal education (66.7%). The majority of key informants were religious (93.3%), whereas a little over half of key informants (53.3%) were currently married. In terms of age, one‐third of key informants (33.3%) were aged between 35 and 54 years, whereas one‐fifth of key informants (20.0%) were aged 55 years or older.

**TABLE 3:** Sociodemographic characteristics of fishers who participated in the key informant interviews (KIIs).

Variable	Category	Frequency (*n*)	Percent
**Sex**	Male	20	66.7
	Female	10	33.3
**Age**	<25	6	20.0
	25–34	8	26.7
	35–44	6	20.0
	45–54	4	13.3
	55–64	3	10.0
	65+	3	10.0
**Education**	No education	10	33.3
	Middle/JHS education	13	43.4
	Secondary/vocational and higher	7	23.3
**Religion**	Islam	3	10.0
	African traditionalist	3	10.0
	No religion	2	6.7
	Christianity	22	73.3
**Marital STATUS**	Never married	8	26.6
	Cohabiting/Informal/Consensual	6	20.0
	Currently married	10	33.3
	Divorced/separated/widowed	6	20.0
**Total**		**30**	**100.0**

### 3.2 Fishers’ Risky Behaviors

The KIIs and FGDs explored participants’ perceptions of risky behaviors among fishers in the Elmina fishing community. Participants perceived three fishers’ behavior as risky: (a) excessive alcohol consumption, (b) excessive smoking, and (c) unsafe sexual practices.

#### 3.2.1 Excessive Alcohol Consumption

Participants in FGDs indicated that excessive alcohol consumption was common among fishers, largely stemming from the prohibition imposed by boat owners against consuming alcohol while at sea. After enduring long hours of work, fishers sought relief through alcohol consumption. Some participants explained that

Many fishing boat owners enforce strict rules that prohibit their crew from consuming alcohol while at sea, aiming to prevent accidents and ensure safety during fishing operations. However, after a long day of hard work, fishers often feel the urge to drink [consume alcohol] to unwind and escape these restrictions, leading to excessive alcohol consumption. [Male FGD P 1].

Tuesdays, when fishers typically do not go out to sea, turn into significant drinking [alcohol consumption] days. These days present opportunities for binge drinking and socializing with friends, allowing fishers to relax and enjoy themselves after the rigors of their work. [Male FGD P 3].

Another participant highlighted the social nature of drinking:

Sometimes, drinking is the only way to feel normal again after many days at sea. It brings us together and helps us forget our problems for a while. [Male FGD P 2].

#### 3.2.2 Excessive Smoking

The FGDs with male and female fishers revealed that smoking was widely adopted as a coping mechanism against the overwhelming stench of the sea, eventually evolving into a regular habit. This theme was consistently reported across both male and female participants, indicating that smoking is a shared coping strategy rather than an isolated behavior. One female participant stated:

The stench of the sea is overwhelmingly strong, often causing many fishers to vomit profusely while out at sea. This unpleasant experience not only affects their physical comfort but also leads them to seek ways to cope with the nausea. As a result, some fishers turn to smoke to mask the odor. [Female FGD P 1].

Another female participant further emphasized the role of smoking in coping with the sea environment:

Sometimes the smell is too much, especially when the fish start going bad. You feel like you cannot even breathe well, so some people smoke to reduce that feeling. [Female FGD P 4].

Some male participants noted:

One way of reducing the effects of the stench is to smoke, and that explains why many of us smoke. Initially used as a coping mechanism to mitigate nausea and discomfort, smoking quickly evolved into a regular practice among many fishers. Over time, the reliance on smoking not only affects our health but also leads to an increased risk of addiction and other related health issues. [Male FGD P 5].

At first, it's just one or two cigarettes, but soon it becomes many in a day. We don't even notice until we can't do without it. [Male FGD P 10].

Another male participant reinforced how smoking becomes normalized over time:

When you are new, you may not smoke, but as time goes on and you keep going to sea, you begin to copy others. Before you realise, it becomes part of your daily routine. [Male FGD P 7].

Similarly, another participant highlighted the social dimension of smoking among fishers:

Most of the guys smoke when we come back from the sea. It becomes something we do together, especially when we are resting and talking. [Male FGD P 8].

#### 3.2.3 Unsafe Sexual Practices

FGDs with female fishers revealed that many of their male partners often refused condom use, especially after consuming alcohol, leading to unsafe sexual encounters. A female fisher shared:

Most of the men I know do not like to use condoms, especially after consuming alcohol. Their reasons are usually based on the belief that ‘raw’ [sex without condom] sex is more enjoyable than one in which a condom is used. [Female FGD P 7].

Participants also indicated that multiple sexual partnerships were common among fishers. Some KII participants narrated that

The male fishers have multiple women on different landing beaches. It's like a normal thing. Some have women in various communities, and they do not hide it. [KII 2].

Because we travel from place to place, we meet many women. Having a woman on every landing site is seen as an achievement by some fishers. [KII 4].

### 3.3 Factors Contributing to Risky Behaviors

This study also explored the factors contributing to risky behaviors among fishers. Three themes emerged as follows: (a) substance abuse leading to inducement, (b) spendthrift habits and lavish social interactions, and (c) perceiving fishing as a risky occupation led to discounting the dangers of HIV.

#### 3.3.1 Substance Abuse Leading to Inducement

All FGDs revealed that substance abuse, particularly the consumption of alcohol and smoking of Indian hemp, is a significant contributor to risky behavior among local fishermen in Elmina and its surrounding areas. Participants indicated that due to excessive alcohol consumption or smoking, many fishers experience impaired judgment and lower inhibitions, leading to engagement in risky sexual behaviors. One participant noted:

Most male fishers consume a lot of alcohol, and when they do, the alcohol lowers their inhibitions, and they end up engaging in risky behavior. When my partner drinks, he never wants to use a condom, and many of the women here will testify that their men are also like that. [KII 3].

Another participant added:

Some male fishers smoke heavily, both cigarettes and Indian hemp, which can lead to a heightened state of intoxication. This altered state of mind often prompts them to engage in risky sexual behaviors, including having multiple sexual partners. [KII 4].

#### 3.3.2 Spendthrift Habits and Lavish Social Interactions

Some participants described a culture among fishers where financial gains from successful catches are frequently squandered on alcohol and women. This behavior reflects the interplay between financial success and social indulgence within the fishing communities. One participant shared:

With a lot of cash on us during this period, we can attract many local women. The money we make from fishing often ends up in the pockets of women and drinking spots where we go to sit and buy drinks. [Male FGD P 7].

Another participant added:

As fishers, when we have money, we spend a lot of it on women and drinks. Often, the local men don't have money to spend on their women, so many women are drawn to us because of our spending habits. [Male FGD P 9].

#### 3.3.3 Perceiving Fishing as a Risky Occupation Led to Discounting the Dangers of HIV

Key informants reported a prevalent perception among fishers that the dangers associated with fishing, such as drowning or facing violent storms at sea, are more immediate and life‐threatening than the risk of HIV infection. Hence, they tend to engage in risky behavior without caution as they perceive their occupational risk as higher than engagement in risky behavior, such as multiple sexual partners and non‐condom use during sexual intercourse, which may heighten their risk of HIV infection. One participant explained that:

There are always risks at sea; it's easy to drown or get caught in strong winds that can flip boats over. Because of this, fishers are constantly put in situations that could kill them every day. This is something that fishers see as more immediate than the fear of HIV and AIDS. [Male FGD P 6].

## 4 Discussion

This study explored fishers’ risky behaviors as well as factors driving these risky behaviors among fishers in Elmina. The findings indicate that fishers engage in excessive alcohol consumption, particularly after work and during days off. This finding is consistent with previous studies [8, 26, 47], which demonstrate that alcohol consumption functions as a coping mechanism for the stress and uncertainty associated with fishing livelihoods. Similarly, Kyei‐Arthur and Kyei‐Gyamfi [4], in a study in Elmina, highlighted that alcohol consumption among coastal workers is driven by both social and functional motivations, facilitating bonding and relaxation in a high‐pressure work environment. These findings should, however, be interpreted within the specific socioeconomic and occupational context of Elmina and comparable fishing communities, rather than generalized to all low‐ and middle‐income country (LMIC) settings.

Although prohibitions established by boat owners effectively reduce accidents at sea, they inadvertently encourage binge drinking onshore. This phenomenon can be explained by RCT, which posits that individuals adjust their behavior in response to perceived changes in risk. The sense of safety provided by maritime prohibitions may lead fishers to engage in riskier behaviors on land, such as excessive alcohol use. Therefore, although such prohibitions are essential for ensuring safety, the integration of post‐work support services, including stress management interventions, is also crucial to mitigate the associated risks. This interpretation reflects behavioral dynamics observed within the Elmina fishing context and may vary across different institutional and cultural settings.

Participants reported that the pervasive stench of the sea contributes to smoking behaviors, a finding corroborated by earlier work [48] and supported by more recent studies showing that environmental stressors and harsh working conditions in fishing communities can facilitate substance use, including smoking, as a coping strategy [3, 8]. The transition from occasional smoking to habitual use observed in this study aligns with the smoking addiction patterns described by Prochaska and Benowitz [49], highlighting how coping strategies can rapidly evolve into health‐compromising habits. Remarkably, the impact of environmental triggers on smoking represents a less explored facet in contemporary literature, thus underscoring a unique occupational health challenge for persons working in the fishing industry. However, such environmental triggers and coping responses are likely to be context‐dependent and should not be assumed to operate uniformly across all fishing populations.

The prevalent refusal to use condoms, particularly following alcohol consumption, mirrors findings from recent studies showing that alcohol use is associated with increased likelihood of unprotected sexual intercourse in high‐risk populations, including fishing communities [3, 8, 50]. The perception that condomless sex is more pleasurable has also been documented in recent research, highlighting persistent barriers to safer sex practices in contexts where cultural norms and substance use influence decision‐making [3, 8, 51].

Importantly, the gender‐segregated FGDs revealed notable differences in how risky sexual behaviors are experienced and negotiated. Female participants emphasized limited power to negotiate condom use, particularly when male partners were intoxicated or controlled financial resources, reflecting gendered power imbalances within transactional sexual relationships. In contrast, male participants often framed multiple partnerships and condom non‐use as normative and socially reinforced behaviors linked to mobility and peer expectations. These findings highlight that risky sexual practices are not only individual choices but are embedded within unequal gender relations that shape agency, negotiation capacity, and exposure to risk. These gendered dynamics are particularly pronounced within the sociocultural setting of Elmina and similar coastal fishing communities, where economic vulnerability, occupational mobility, and gendered divisions of labor shape relationship dynamics and constrain women's ability to negotiate safer sexual practices [18, 52, 53].

Moreover, the tendency for fishers to engage in multiple concurrent partnerships aligns with recent studies in fishing communities in sub‐Saharan Africa, which show that mobility, transactional relationships, and social norms contribute to increased vulnerability to STIs [3, 8]. In this study, fishers’ perception of fishing as a high‐risk occupation further exemplifies RCT; the constant exposure to immediate life‐threatening dangers at sea may lead to the undervaluation of longer term health threats like HIV/AIDS. Feeling that daily survival is already precarious, fishers may unconsciously rationalize engaging in risky sexual behaviors, viewing them as less significant compared to occupational hazards. This pattern reflects locally embedded perceptions of risk and livelihood realities [8, 52], and caution should be exercised in extending these interpretations beyond similar fishing contexts.

The findings of this study illuminate an array of interrelated factors that contribute to risky behaviors among fishers in Elmina, prominently featuring substance abuse, spendthrift habits associated with lavish social interactions, and the perception of fishing as an intrinsically perilous occupation. The role of substance abuse in facilitating risky sexual behavior among fishers is well‐documented. Numerous recent studies have shown that alcohol consumption, smoking, and drug use impair judgment and reduce inhibitions, thereby increasing the likelihood of engaging in risky sexual behaviors, such as unprotected sex and multiple partnerships [4, 26, 54, 55, 56]. Although similar patterns have been reported in other fishing communities in sub‐Saharan Africa, where mobility, economic vulnerability, and gendered power relations shape risky behaviors [3, 21], the present study provides context‐specific insights that should be interpreted within the sociocultural and economic setting of Elmina [4, 18, 53].

### 4.1 Limitations and Strengths of the Study

This study has some limitations. First, the study was cross‐sectional, so it does not allow for an understanding of changes in fishers’ risky behavior over time. In addition, the cross‐sectional design does not allow for causal inferences; therefore, the findings should be interpreted as indicating associations and context‐specific explanations of behavior rather than cause‐and‐effect relationships. Future research using longitudinal designs is recommended to better capture causal pathways and changes in behavior over time. Second, the responses of participants were self‐reported. As risky behaviors are perceived as negative behaviors, there is a possibility that participants may provide socially desirable responses. To mitigate this limitation, the study employed data triangulation across multiple qualitative sources, including KIIs and FGDs, and cross‐checked responses for consistency across participants.

Third, the study was conducted in Elmina. Hence, extensive replication is required before it can be generalized to other fishing communities. Relatedly, the focus on a single fishing community with a relatively limited sample size may constrain the representativeness of the findings and limit their external validity across diverse fishing contexts in Ghana. As such, the findings should be interpreted as context‐specific rather than nationally representative.

Additionally, the data for this study were collected in 2017. Although the core structural conditions of fishing communities (e.g., mobility, livelihood patterns, and social dynamics) remain largely consistent, it is possible that changes in policies, health interventions, and social behaviors over time may affect the current applicability of the findings. This potential time lag should therefore be considered when interpreting the results.

Despite these limitations, the study contributes to the literature on risky behaviors among fishers and the factors contributing to these behaviors. Notably, the study provides rich, in‐depth qualitative insights drawn from multiple data sources, enhancing the credibility and depth of the findings. Furthermore, by applying RCT in a more analytically grounded manner, the study offers a nuanced understanding of how fishers interpret and respond to competing risks within HIV hotspot communities. These strengths make the study a valuable contribution to ongoing efforts to design context‐specific interventions in fishing communities.

### 4.2 Implications for Policy and Practice

The findings of the study present several significant implications for policy and practice. Interventions should extend beyond individual behavioral modifications to address structural and environmental determinants. Policies designed to mitigate substance abuse among fishers must include community‐driven awareness initiatives, peer support networks, and rehabilitation services that are adapted to the specific circumstances of fishing life. To enhance implementation, these interventions can be operationalized through collaboration between the Ministry of Health, the Ghana AIDS Commission, local government authorities, and fisher associations. For example, community‐based peer educators can be trained within fishing associations to deliver regular sensitization sessions, whereas local health facilities can provide mobile outreach services during peak fishing periods.

Furthermore, programs ought to incorporate financial management education to address the spendthrift culture identified in the study. Equipping fishers with financial literacy skills has the potential to alter social norms, promoting sustainable financial behaviors over extravagant spending, which may subsequently decrease risky sexual encounters. Such programs can be integrated into existing community structures, such as fisher cooperatives and landing site associations, with support from financial institutions and NGOs to deliver practical training on savings, investment, and income management.

Moreover, health education strategies should specifically target the distorted risk perceptions associated with the hazardous nature of fishing. HIV prevention campaigns must reframe the narrative to emphasize that although occupational hazards are immediate, the gradual progression of HIV infection does not lessen its severity. Effective risk communication strategies should be culturally relevant and incorporate the involvement of local leaders and fisherfolk in both advocacy and design processes. Implementation can involve the co‐creation of messages with community leaders and the use of local languages and storytelling approaches during community gatherings and festivals to improve acceptance and effectiveness.

Establishing secure recreational areas and offering alternative social opportunities for fishers during periods of inactivity may substantially diminish dependence on alcohol and casual sexual encounters as predominant leisure activities. These spaces can be developed through partnerships between district assemblies, private sector actors, and community groups, with clear management structures to ensure sustainability and accessibility. Adapting interventions to the specific contexts and dynamics of fishing communities is crucial for ensuring their relevance, acceptance, and sustainability over time.

Overall, a multi‐stakeholder approach with clearly defined roles, community ownership, and integration into existing local systems is essential for the feasibility and long‐term success of these interventions.

## 5 Conclusion

This study sheds light on the complex web of factors driving risky behaviors among fishers in the Elmina community. Substance abuse, spendthrift social patterns, and occupational risk perceptions emerged as critical contributors to unsafe sexual practices. The application of RCT revealed that the measures intended to enhance safety, such as prohibitions against drinking at sea, may inadvertently push fishers toward greater risk‐taking onshore. Furthermore, the normalization of immediate life‐threatening dangers associated with fishing appears to reduce the perceived severity of health risks like HIV/AIDS, reinforcing neglect of safe sex practices. The findings underscore the need for holistic interventions that address not just individual behaviors but also the broader environmental, occupational, and socioeconomic contexts that shape the decision‐making of fishers. Importantly, substance abuse prevention, financial literacy training, and reshaping risk perceptions are crucial components of any effective intervention. Future research should further investigate how occupational culture influences health behavior and explore sustainable models for behavior change within fishing communities.

## Author Contributions

Sylvester Kyei‐Gyamfi contributed to the conceptualization and design of the study. Frank Kyei‐Arthur, Sylvester Kyei‐Gyamfi, Oppong Kwarteng Hammond, Bismark Bless Nyadzi, Joha Issaka Braimah, Eunice Kisiwaa Gyan analyzed the data. Frank Kyei‐Arthur, Sylvester Kyei‐Gyamfi, Oppong Kwarteng Hammond, Bismark Bless Nyadzi, Joha Issaka Braimah, and Eunice Kisiwaa Gyan wrote the first draft of the manuscript. Finally, Frank Kyei‐Arthur, Sylvester Kyei‐Gyamfi, Oppong Kwarteng Hammond, Bismark Bless Nyadzi, Joha Issaka Braimah, and Eunice Kisiwaa Gyan contributed to editing the manuscript and reading and approving the final version submitted.

## Funding

The authors have nothing to report.

## Ethics Statement

This study was approved by the Ethics Committee for the Humanities at the University of Ghana (ECH 118/16‐17). Participation was voluntary, and all participants provided written informed consent prior to data collection. The study involved adult participants (18 years and above), including fishers, community members, and key informants from government institutions, NGOs, and local leadership within the Elmina fishing community. Socio‐demographic characteristics of participants are presented in the Results section.

## Consent

The authors have nothing to report.

## Conflicts of Interest

The authors declare no conflicts of interest.

## Data Availability

The datasets generated and/or analyzed during the current study are available from the corresponding at reasonable request.
